# Long-Term Treatment with Fluvoxamine Decreases Nonmotor Symptoms and Dopamine Depletion in a Postnatal Stress Rat Model of Parkinson's Disease

**DOI:** 10.1155/2020/1941480

**Published:** 2020-03-20

**Authors:** Ernest Dallé, William M. U. Daniels, Musa V. Mabandla

**Affiliations:** ^1^School of Laboratory Medicine and Medical Sciences, College of Health Sciences, University of KwaZulu-Natal, Durban 4000, South Africa; ^2^Faculty of Medicine and Biomedical Sciences, Yaoundé I, Yaoundé, Cameroon; ^3^School of Physiology, University of the Witwatersrand, Johannesburg, South Africa

## Abstract

Nonmotor symptoms (NMS) such as anxiety, depression, and cognitive deficits are frequently observed in Parkinson's disease (PD) and precede the onset of motor symptoms by years. We have recently explored the short-term effects of Fluvoxamine, a selective serotonin reuptake inhibitor (SSRI) on dopaminergic neurons in a parkinsonian rat model. Here, we report the long-term effects of Fluvoxamine, on early-life stress-induced changes in the brain and behavior. We specifically evaluated the effects of Fluvoxamine on brain mechanisms that contribute to NMS associated with PD in a unilateral 6-hydroxydopamine-lesioned rat model. A 14-day early postnatal maternal separation protocol was applied to model early-life stress followed by unilateral intracerebral infusion of 6-hydroxydopamine (6-OHDA) to model aspects of parkinsonism in rats. The anxiolytic, antidepressant, and cognitive effects of Fluvoxamine were confirmed using the elevated plus-maze (EPM) test, sucrose preference test (SPT), and Morris water maze (MWM) test. Further to that, our results showed that animals exposed to early-life stress displayed increased plasma corticosterone and malondialdehyde (MDA) levels which were attenuated by Fluvoxamine treatment. A 6-OHDA lesion effect was evidenced by impairment in the limb-use asymmetry test as well as decreased dopamine (DA) and serotonin levels in the striatum, prefrontal cortex, and hippocampus. These effects were surprisingly attenuated by Fluvoxamine treatment in all treated rats. This study is the first to suggest that early and long-term treatment of neuropsychological diseases with Fluvoxamine may decrease the vulnerability of dopaminergic neurons that degenerate in the course of PD.

## 1. Introduction

Chronic stress can promote or even cause psychological disorders such as anxiety, depression, and cognitive deficits which are, in fact, the results of a blunted hypothalamic-pituitary-adrenal (HPA) axis response and altered neurotransmitter levels [1–3]. Besides, stress has been shown to have a major impact on neurodegeneration affecting behavioral symptoms in brain disorders such as Parkinson's disease (PD) [3, 4]. There is considerable evidence to show that most individuals with PD also display anxiety, depression, or cognitive deficits many years before their diagnosis of PD [5–9]. It is well known that exposure to stress during early life can have long-term detrimental effects on brain development [1]. The chronic stress caused by social isolation early in life contributes to the learning and memory performance and psychological disorders such as generalized anxiety and depression in a developing brain, which often prompts motor dysfunction characteristic of PD [3, 10–12].

Fluvoxamine is a selective serotonin reuptake inhibitor (SSRI) well known for its efficacy in treating major depression [13]. This antidepressant is safe and highly effective compared to other antidepressants such as fluoxetine, imipramine, and chlorimipramine [14, 15]. Fluvoxamine works by improving the clinical profile supposedly influencing serotonin levels in patients with depression [16]. However, besides altered serotonin levels, Fluvoxamine has also been shown to increase the release of dopamine (DA) which is known to be decreased in patients with PD [17–19]. It is therefore suggested that Fluvoxamine treatment influences both the serotonergic and the dopaminergic systems in terms of its mechanism of action [19, 20].

To develop better therapeutic strategies, these must first be tested in animal models as they are of paramount importance in obtaining insight into the pathogenesis of all diseases [7, 21]. Animal models of neuropsychiatric and/or neurodegenerative diseases are well-established and have been extensively used to understand the pathogenesis of diseases as well as to find therapeutic strategies to combat the disease [22–24]. However, to date, there is no proven therapy that can prevent cell death, slow disease progression, or restore healthy function in diseased neurons [24]. To create an animal model of early stress, two weeks of maternal separation during the stress-hyporesponsive period (early postnatal period) has been shown to impair the development of brain neural circuitry leading to disruption of the hippocampal cytoarchitecture [12, 25–27]. Also, an intracerebral injection of the neurotoxin 6-hydroxydopamine (OHDA) into the medial forebrain bundle (MFB) has been shown to result in a parkinsonian rat model [28, 29]. A combined model of early stress and neurodegeneration is therefore useful to evaluate the effects of a pharmacological agent in ameliorating nonmotor symptoms (NMS) associated with PD. The present study is therefore aimed at showing the long-term effects of Fluvoxamine on brain mechanisms that contribute to NMS (anxiety/depressive-like behavior and cognitive deficits) associated with PD development.

## 2. Materials and Methods

**Figure 1:**
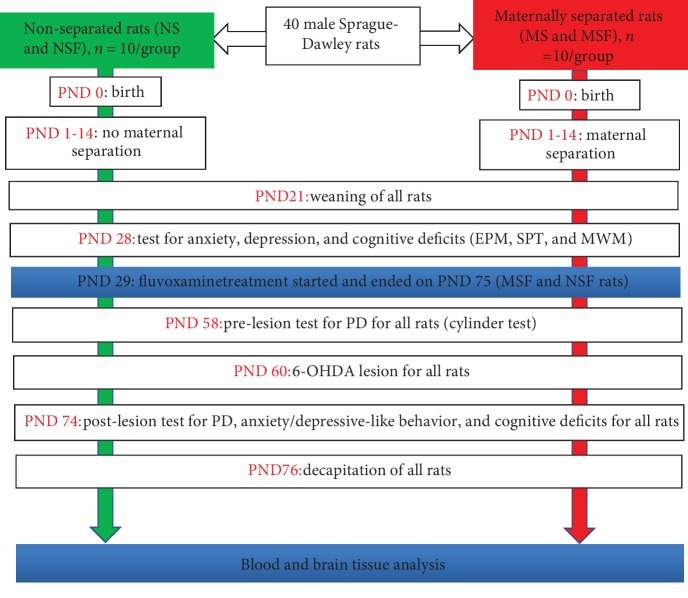
Diagram summarizing the experimental protocols. NS: nonseparated lesioned rats; NSF: nonseparated lesioned rats treated with Fluvoxamine from PND 29-75; MS: maternally separated lesioned rats; MSF: maternally separated lesioned rats treated with Fluvoxamine from PND 29-75; EPM: elevated plus maze; SPT: sucrose preference test; MWM: Morris water maze; PND: postnatal day.

The experimental protocol (Figure 1) used for this work was approved (ref: 061/14/Animal) by the University of KwaZulu-Natal Animal Research Ethics Committee, and the study was conducted in accordance with the National Institutes of Health requirements for the care and use of laboratory animals.

### 2.1. Drugs, Reagents, and Method of Determination

Fluvoxamine (ref: 633956), Temgesic (ref: 5591), and Biotaine (ref: 0501291) were purchased from Pharmed Pharmaceuticals LTD (Rochdale Park, Durban, South Africa). Desipramine (ref: D3900), atropine (ref: 106026), pentobarbital (ref: 102038), and 6-OHDA (ref: MKBX4996V) were purchased from Sigma-Aldrich (St. Louis MO, USA). The corticosterone ELISA kit (ref: RE52211), the dopamine ELISA kit (ref: RE59161), and the serotonin ELISA kit (ref: RE59121) were all purchased from IBL International GmbH (Hamburg, Germany). The malondialdehyde (MDA) assay kit (ref: K739-100) was from BioVision (Centurion, South Africa). The method of determination of all the assays used in this study was based on the enzyme-linked immunosorbent assay (ELISA) methods that target antigen (or antibody) capture in samples using a specific antibody (or antigen) and target molecule detection/quantitation using an enzyme reaction with its substrate. This simple and highly sensitive spectrophotometric method was used for the determination of corticosterone, malondialdehyde (MDA), dopamine, and serotonin levels in blood and brain tissues.

### 2.2. Animals

A total of 40 male Sprague-Dawley (SD) rats, weighing between 6 g at postnatal day (PND) 1 and 300 g at PND 76, was used. Pregnant SD rats were obtained from the Biomedical Resource Unit of the University of KwaZulu-Natal and were housed in polypropylene cages (38 × 32 × 16 cm) under controlled temperature (21 ± 2°C) and humidity (55–60%) until they gave birth [30]. At birth, pups were crossfostered and randomly divided into 4 groups as follows: (1) nonmaternally separated (NS), (2) nonmaternally separated treated with Fluvoxamine (NSF), (3) maternally separated (MS), and (4) maternally separated treated with Fluvoxamine (MSF) (*n* = 10/group). Food and water were freely available, and bedding was changed twice a week. After weaning (PND 21), animals were transferred from the polypropylene cages to transparent glass cages and kept six per cage under a standard daily light/dark circle (lights on at 06:00 am until 06:00 pm) [31]. Animals were weighed daily before all laboratory experiments and were brought to the experimental room at least one hour prior to the beginning of any experiment or decapitation. All animals were given 1 hour to rest between two consecutive experimental procedures. Apparatus and instruments used during experiments, surgery, or treatments were systematically cleaned with an antiseptic (Biotaine) before and after each experiment and between 2 animals being tested or simply replaced.

### 2.3. Methods

Both the pups and their dams were housed in transparent glass cages of 40 cm length, 20 cm width, and 24 cm height. Experimental pups were exposed to a chronic stress experience of being separated from their dam for a 3-hour period daily for 14 days.

### 2.4. Stress Protocol

Maternal separation was implemented from PND 1-14. The maternal separation stress protocol consisted of removing the dams from their respective cages and taking them into another room, leaving the pups alone for 3 hours daily from 9:00 am to 12:00 pm [29, 32].

### 2.5. Fluvoxamine Treatment Regimen

The Fluvoxamine tablets were crushed then dissolved in saline before the daily intraperitoneal injection (25 mg/kg, i.p.) [33]. The injected Fluvoxamine solution was always freshly prepared on the day of treatment.

### 2.6. Behavioral Tests for Anxiety/Depressive-Like Behavior and Cognitive Deficits

Anxiety/depressive-like behavior and cognitive deficits were assessed on PND 28 and PND 74 using the elevated plus-maze (EPM) test, the sucrose preference test (SPT), and the Morris water maze (MWM) test.

#### 2.6.1. Elevated Plus-Maze (EPM) Test

The EPM test was used to evaluate anxiety-like behavior in rats [34–36]. The maze consisted of four arms (two open without walls and two enclosed by 15.25 cm high walls) 30 cm long and 5 cm wide [37]. Each arm of the maze was attached to sturdy wood legs such that it was elevated 40 cm above the floor [37]. Each rat was individually given a 5-minute trial in the apparatus, and the time spent by the animals in the open and closed arms of the maze was scored [38]. Indices of anxiety-like behavior were a significantly elevated time spent on the closed arms or a significantly reduced time spent on the open arms [27, 34, 36, 39].

#### 2.6.2. Sucrose Preference Test (SPT)

The individual housing for the SPT began on PND 27 (24 hours before the actual test). The SPT was conducted over a 24-hour period on PND 28 and PND 74 to evaluate anhedonia in our rats as described by Dalle et al. [19]. Briefly, the test consisted of placing two bottles (one containing tap water and the other containing 2.5% sucrose solution) in each animal's cage, to evaluate the consumption/preference for water and/or sucrose. The bottles were weighed 24 hours later to determine the consumption in grams (converted to ml), and the sucrose preference ratio was calculated as the volume of sucrose drunk divided by the total fluid consumed (sucrose+water). A reduced preference for the sucrose solution was suggestive of anhedonia thus of depressive-like behavior [40–43].

#### 2.6.3. Morris Water Maze (MWM)

The MWM test was used to test spatial learning and memory, an important cognitive component of neurodegenerative diseases including Parkinson's disease [44, 45]. The MWM apparatus was a circular tank (1 m in diameter, divided into 4 quadrants) filled with water (22-23°C) in which a hidden square plexiglass platform (10 × 10 cm wide and 20 cm high) was submerged (1 mm) below the water surface. The test conducted on PND 28 briefly consisted of a 2-minute repeated trials whereby the rat was placed in the tank and time taken (latency) to find the hidden platform was recorded as the learning (acquisition) process [46]. The animal learned to locate the platform using visual cues placed above the quadrant of the tank and visible to the swimming rat. A probe test (without the platform) conducted on PND 74 assessed the memory retrieval of the rats to remember the quadrant in which the platform was located [47]. The time the animal spent investigating the area of the tank that formerly contained the platform was recorded.

### 2.7. Stereotaxic Injection of 6-Hydroxydopamine (6-OHDA)

On PND 60, thirty minutes prior to the 6-OHDA infusion, desipramine HCl (15 mg/kg), a norepinephrine transporter blocker, was injected intraperitoneally to protect noradrenergic neurons from damage by the neurotoxin 6-OHDA [48–50]. Animals were anesthetized with sodium pentobarbital (60 mg/kg, i.p.) followed by an injection of atropine (0.2 mg/kg, i.p.). After full anesthesia was confirmed, the head of the animal was shaved and firmly positioned in the stereotaxic apparatus (David Kopf Instruments, Tujunga, CA, USA). The skin covering the scalp was disinfected with an antiseptic solution, and a midline incision was made to expose the skull. A hole was drilled in the skull according to Mabandla and Russell [29] at the following coordinates: anterior − posterior (AP) = +4.7 mm anterior to lambda; mediolateral (ML) = +1.6 mm lateral to midline suture; and DV = −8.4 mm ventral to dura. At these coordinates, a 10 *μ*l Hamilton microsyringe with a 32-gauge needle was used to slowly inject 6-OHDA (5 *μ*g/4 *μ*l) dissolved in saline containing 0.2% ascorbic acid into the left medial forebrain bundle (MFB) over a period of 8 minutes at a rate of 0.5 *μ*l/min. The infusion needle was kept in the MFB for 1 minute prior to the injection and an additional two minutes was allowed to maximize the diffusion of the drug before the needle was slowly removed from the brain. The wound was sutured and cleaned with Biotaine, and the animals were then placed under a heating lamp for further observation. Temgesic (0.05 mg/kg, subcutaneously), as a postoperative analgesic, was administered to each animal before being returned to their housing room.

### 2.8. Behavioral Test for Parkinsonism

The limb-use asymmetry test (cylinder test) was used to provide information about preferred use of the unimpaired limb (ipsilateral to the 6-OHDA injection site) while performing weight-shifting movements (touching the wall of the cylinder and wall exploration while standing on its hind limbs and when landing). A prelesion test was performed at PND 58 and a postlesion test at PND 74 for comparative purposes to evaluate a possible attenuation of locomotor deficits caused by treatment with Fluvoxamine. The limb-use asymmetry apparatus was similar to the one described by Mabandla and Russell [29] and Meredith and Kang [51] and consisted of a transparent plexiglass cylinder, 20 cm in diameter and 30 cm in height. The animal was placed in the cylinder and its behavior recorded for a 5-minute trial using a video camera. The forelimb used during exploratory activity was scored. As the neurotoxin 6-OHDA was infused into the left hemisphere only, a successful unilateral model of parkinsonism was expected to prefer to use its left forelimb after the lesion compared to before the lesion [29, 52].

### 2.9. Decapitation

All animals were sacrificed on PND 76 using a sharp guillotine [53]. Blood and brain tissues (striatum, hippocampus, and prefrontal cortex) were collected after decapitation. A maximum of 5 ml of blood was collected, immediately dispensed into precooled heparinized tubes, and centrifuged at 1322 × g for 10 minutes at 4°C using a refrigerated centrifuge (Labofuge 200, Heraeus Sepatech, Hanau, Germany). The plasma was thereafter collected in Eppendorf tubes (1.5 ml), sealed with Parafilm, snap frozen in liquid nitrogen, and stored in a biofreezer at -80°C until the day of analysis. As per Glowinski et al. [54], the whole brain was manually removed from the skull and the hippocampus, striatum, and prefrontal cortex were dissected immediately on an ice-cooled glass slab, weighed, put into Eppendorf tubes, sealed with Parafilm, snap frozen in liquid nitrogen, and thereafter stored in a biofreezer at -80°C until the day of the assay.

### 2.10. Biochemical Analyses

#### 2.10.1. Corticosterone

A volume of 20 *μ*l of each standard, control, and plasma sample was dispensed into the appropriate wells of a microtiter plate. Enzyme conjugate (200 *μ*l) was added into all wells, mixed, and incubated for an hour at room temperature. Substrate solution (100 *μ*l) supplied with the assay kit was thereafter added, and 15 minutes was allowed for incubation at room temperature. Stop solution (50 *μ*l) supplied with the assay kit was added after the incubation period, and the optical density of standards, controls, and samples was measured using a microtiter plate reader (SPECTROstar Nano, BMG LABTECH GmbH, Ortenberg, Germany) at 450 nm within 10 minutes as per the manufacturer's protocol of the assay kit. A standard curve was generated with known concentration of analytes and used for quantitative analysis.

#### 2.10.2. Lipid Peroxidation Analysis

Malondialdehyde (MDA) as a lipid peroxidation marker was measured (according to the manufacturer's protocol of the assay kit) in the dorsal hippocampus, striatum, and prefrontal cortex of the left hemisphere to quantify oxidative stress. Brain tissue was homogenized with a sonicator (CML-4, Fisher, USA) on ice in 300 *μ*l of the MDA lysis buffer, then centrifuged at 17761 × g for 10 minutes. A volume of 200 *μ*l of the supernatant from each homogenized sample was thereafter placed into a microcentrifuge tube. To prepare standards, 10 *μ*l of MDA standard was diluted with 407 *μ*l of bidistilled water to prepare a solution of 0.1 M MDA; then, 20 *μ*l of 0.1 M MDA solution was diluted with 980 *μ*l of bidistilled water to prepare a 2 mM MDA standard. Into standard and sample vials, 600 *μ*l of thiobarbituric acid (TBA) solution was added and incubated for 1 hour in a water bath at 95°C. Standard and control vials were allowed to cool at room temperature for 10 minutes, and thereafter, 200 *μ*l from each standard and sample was pipetted in duplicate into a 96-well microplate for analysis according to the manufacturer's protocol. Lipid peroxidation was determined by estimating the level of thiobarbituric acid-reactive substances (TBARS) measured as malondialdehyde (MDA), according to the method used by Hall and Bosken [55].

#### 2.10.3. Dopamine (DA)

The level of DA was measured in the left hemisphere striatum, prefrontal cortex, and dorsal hippocampus using a dopamine ELISA kit according to the manufacturer's instructions. The protocol consisted of an extraction of samples and standards, followed by a quantification procedure the next day where the optical density of extracted samples and standards was measured at 450 nm using a spectrophotometer. Briefly, brain tissue was thawed at room temperature and then, 500 *μ*l of EDTA-HCl buffer was added into each Eppendorf tube containing the tissue. The tissue was homogenized with a sonicator (CML-4, Fisher, USA) and thereafter centrifuged at 1287 × g for 10 minutes at 4°C. The supernatant was pipetted into a new Eppendorf tube. For the extraction, 20 *μ*l of each standard and control was pipetted into the respective extraction plate wells. A volume of 500 *μ*l of each sample was also pipetted into the remaining extraction plate wells. Standards and controls were diluted with 500 *μ*l of bidistilled water to correct for the differences in volume according to the manufacturer's instruction. For the quantification, 50 *μ*l of release buffer was added to extracted standards and controls. Thereafter, 100 *μ*l of prediluted standards and controls and 100 *μ*l of extracted samples (without predilution) were pipetted into respective wells containing 75 *μ*l of COMT enzyme solution that was supplied with the kit according to the manufacturer's protocol. The analysis was performed in duplicate. The optical density of the samples was read within 10 minutes after the stop solution was added using a microtiter plate reader (SPECTROstar Nano, BMG LABTECH GmbH, Ortenberg, Germany) set at 405 nm.

#### 2.10.4. Serotonin

Brain tissues were homogenized with a sonicator (CML-4, Fisher, USA) and thereafter centrifuged at 340 × g at 4°C for 10 minutes according to the manufacturer's instructions. A volume of 250 *μ*l of 0.05 M HCl containing 0.1% ascorbic acid was added to the homogenized tissue, shaken (protected from sunlight) for an hour, and then centrifuged for 20 minutes at 1681 × g. The supernatant of each sample was then collected into new Eppendorf tubes. Twenty-five (25) *μ*l of the standard, control, and sample was pipetted into reaction tubes provided with the kit. A volume of 500 *μ*l of acylation buffer supplied with the kit and 25 *μ*l of acylation reagent supplied with the kit was added into all tubes, mixed, and incubated for 15 minutes at room temperature. A volume of 25 *μ*l of the acylated standard, control, and sample was pipetted into respective wells of the serotonin microtiter strips (provided with the kit). A volume of 100 *μ*l of the serotonin antiserum was pipetted into each well and incubated for 30 minutes, and the content of each well was washed with 300 *μ*l of wash buffer and dried on paper towel. Thereafter, 100 *μ*l of conjugate was pipetted into each well and allowed to incubate for 15 minutes and the content discarded. The well was washed and dried on a paper towel. A volume of 100 *μ*l of the substrate supplied with the kit was pipetted into each well and allowed to incubate for 15 minutes, and 100 *μ*l of the stop solution supplied with the kit was added into each well. The absorbance of the solution in the wells was read within 10 minutes using a microplate reader set at 450 nm.

### 2.11. Statistical Analysis

Results are presented as mean ± SEM. The data were analyzed using the software program GraphPad Prism (version 8, San Diego, California, USA). D'Agostino-Pearson omnibus normality test was used to test the distribution of our data, and parametric tests were used to analyze them. The unpaired *t*-test was used when only comparing the mean of 2 groups (NS versus MS) on PND 28 in the SPT and in the MWM test. Repeated measures analysis of variance (ANOVA) was used for the EPM analysis on PND 28 to compare the time spent in the open and closed arms. In the EPM on PND 74, three-way ANOVA followed by Tukey's post hoc test was used where stress, Fluvoxamine treatment, and the arm preference were the 3 main factors considered. In SPT, MWM, cylinder test, MDA, DA, and serotonin analysis, two-way ANOVA followed by Tukey's post hoc test was used considering stress and Fluvoxamine treatment as the 2 main factors on PND 28 and 74. *p* < 0.05 was considered significant in all analyses.

## 3. Results

### 3.1. Plasma Corticosterone Levels

**Figure 2:**
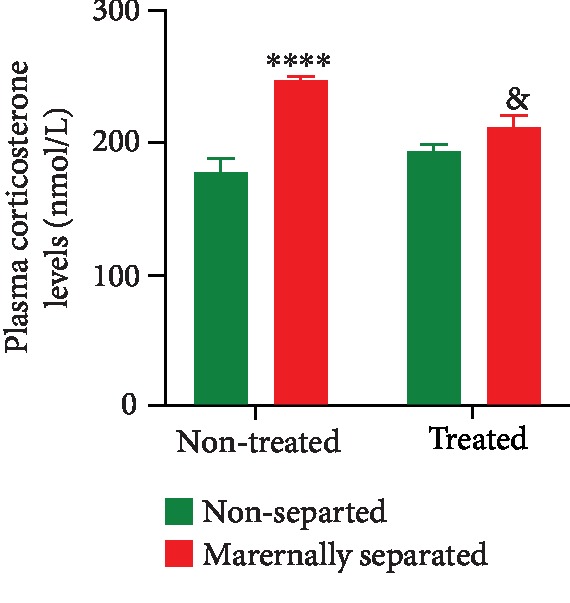
Effect of Fluvoxamine on plasma corticosterone levels of nonseparated (NS, NSF) and maternally separated (MS, MSF) rats at PND 76. A two-way ANOVA revealed a significant main effect of stress (*F*_(1, 36)_ = 33.25, *p* < 0.0001; ^∗∗∗∗^NS vs. MS, *p* < 0.0001, Tukey post hoc test) and a significant interaction between stress and Fluvoxamine treatment (*F*_(1, 36)_ = 11.57, *p* = 0.0017; ^&^MS vs. MSF, *p* = 0.0106, Tukey post hoc test). Data are expressed as mean ± SEM (*n* = 10/group).

Plasma corticosterone levels were measured on PND 76 in all rats. A two-way ANOVA revealed a significant main effect of stress (*p* < 0.0001). The plasma corticosterone levels of maternally separated rats were higher than those of the nonseparated rats (NS vs. MS, *p* < 0.0001). A two-way ANOVA revealed a significant interaction between stress and Fluvoxamine treatment (*p* = 0.0017). Fluvoxamine treatment decreased plasma corticosterone levels in maternally separated rats (MS vs. MSF, *p* = 0.0106). Data are presented in Figure 2.

### 3.2. Elevated Plus Maze (EPM)

**Figure 3:**
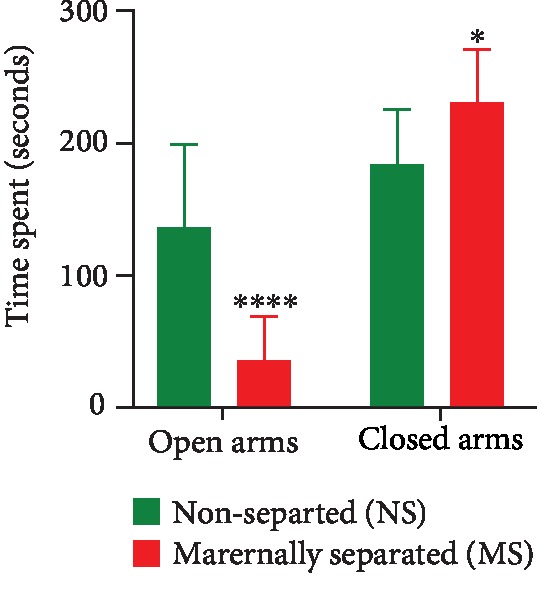
Time spent in the open and closed arms of the elevated plus maze as a measure of anxiety-like behavior on PND 28. Two-way repeated measures ANOVA revealed significant effects of stress (*F*_(1, 22)_ = 8.167, *p* = 0.0091) and arm preference (*F*_(1, 22)_ = 57.03, *p* < 0.0001) and a significant interaction between stress and arm preference (*F*_(1, 22)_ = 21.14, *p* = 0.0001). ^∗∗∗∗^Open NS vs. open MS, *p* < 0.0001 and ^∗^closed NS vs. closed MS, *p* = 0.0322, Tukey post hoc test. Data are presented as mean ± SEM (*n* = 20/group).

On PND 28, nonseparated (NS) and maternally separated (MS) rats were assessed for anxiety-like behavior in the EPM. Repeated measures two-way ANOVA showed a significant main effect of stress (*p* = 0.0091) and arm preference (*p* < 0.0001), as well as an interaction between stress and arm preference (*p* = 0.0001). NS rats spent significantly more time in the open arms of the EPM than MS rats (open NS vs. open MS, *p* < 0.0001). Stress decreased time spent in the open arms and increased time spent in the closed arms (closed NS vs. closed MS, *p* = 0.0322). The data are presented in Figure 3.

**Figure 4:**
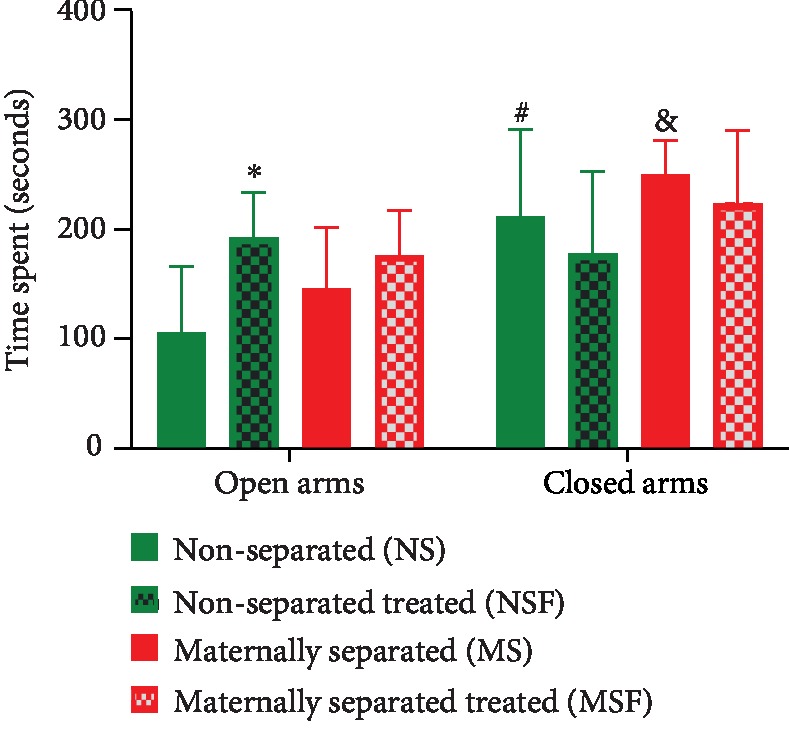
Effect of Fluvoxamine treatment on the time spent by nonseparated (NS, NSF) and maternally separated (MS, MSF) rats in the open and closed arms of the elevated plus maze on PND 74. ^∗^Open NS vs. open NSF, *p* = 0.0186; ^#^open NS vs. closed NS, *p* = 0.0021; ^&^open MS vs. closed MS, *p* = 0.0026, Tukey post hoc test. Data are presented as means ± SEM (*n* = 10/group).

On PND 74, all nontreated (NS, MS) and treated (NSF, MSF) rats were reassessed for anxiety-like behavior in the EPM. Three-way ANOVA revealed significant arm preference effect (*p* < 0.0001) and a significant interaction between Fluvoxamine treatment and arm preference (*p* = 0.0012). Tukey post hoc test revealed a significant difference between open NS vs. open NSF (*p* = 0.0186), open NS vs. closed NS (*p* = 0.0021), and open MS vs. closed MS (*p* = 0.0026). Data are presented in Figure 4.

### 3.3. Sucrose Preference Test (SPT)

**Figure 5:**
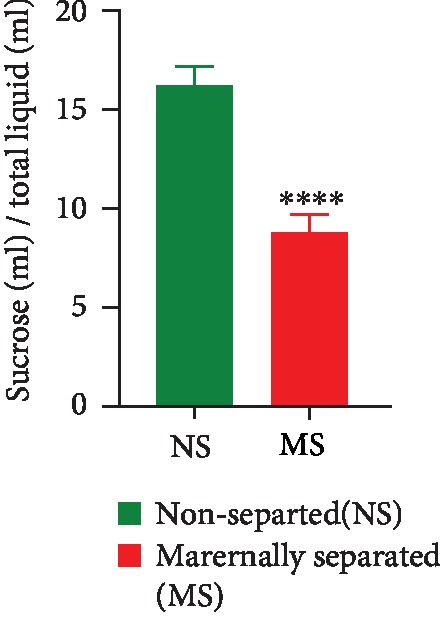
Sucrose consumption in all rats assessed on PND 28. ^∗∗∗∗^NS vs. MS, *p* < 0.0001, unpaired *t*-test. Values are expressed as mean ± SEM (*n* = 20/group).

In the SPT conducted on PND 28, there was a stress effect observed as nonseparated (NS) rats consumed significantly less sucrose solution than maternally separated (MS) rats (NS vs. MS, *p* < 0.0001). Data are presented in Figure 5.

**Figure 6:**
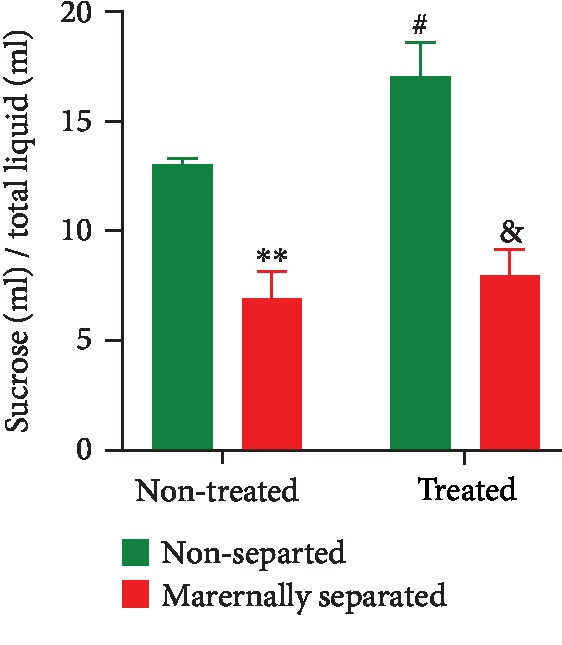
Effect of Fluvoxamine treatment on sucrose consumption by nonseparated (NS, NSF) and maternally separated (MS, MSF) rats at PND 74. Two-way ANOVA revealed an overall effect of stress (*F*_(1, 36)_ = 43.74, *p* < 0.0001; ^∗∗^NS vs. MS, *p* = 0.0032; ^&^NSF vs. MSF, *p* < 0.0001) and Fluvoxamine treatment (*F*_(1, 36)_ = 4.79, *p* = 0.0352; ^#^NS vs. NSF, *p* = 0.0175, Tukey post hoc test). Values are expressed as mean ± SEM (*n* = 10/group).

On PND 74, a two-way ANOVA revealed a stress effect (*p* < 0.0001). Maternally separated (MS) rats consumed less sucrose solution than nonseparated (NS) rats. There was also an overall Fluvoxamine treatment effect (*p* = 0.0352). Fluvoxamine treatment resulted in an increase in sucrose consumption in nonseparated treated rats (^#^NS vs. NSF, *p* = 0.0175). Tukey post hoc test showed that MS rats consumed less sucrose solution than NS rats (NS vs. MS, *p* = 0.0032) and nonseparated rats treated with Fluvoxamine consumed more sucrose solution than maternally separated rats treated with Fluvoxamine (NSF vs. MSF, *p* < 0.0001). Data are presented in Figure 6.

### 3.4. Morris Water Maze (MWM) Test

**Figure 7:**
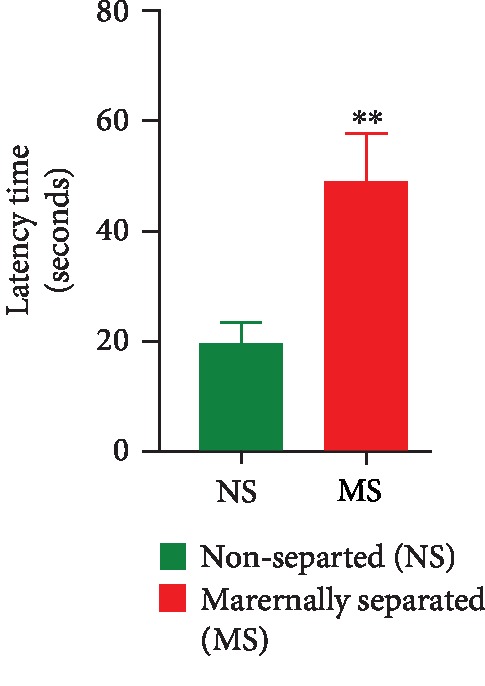
Time taken by nonseparated (NS) and maternally separated (MS) rats to find the hidden platform in the MWM as a measure of their learning ability. ^∗∗^NS vs. MS, *p* = 0.0037, unpaired *t*-test. Data are presented as mean ± SEM (*n* = 20/group).

The learning ability and memory retrieval were assessed on PND 28 and 74, respectively. On PND 28, there was a significant stress effect as maternally separated (MS) rats took longer to find the platform (NS vs. MS, *p* = 0.0037). Data are shown in Figure 7.

**Figure 8:**
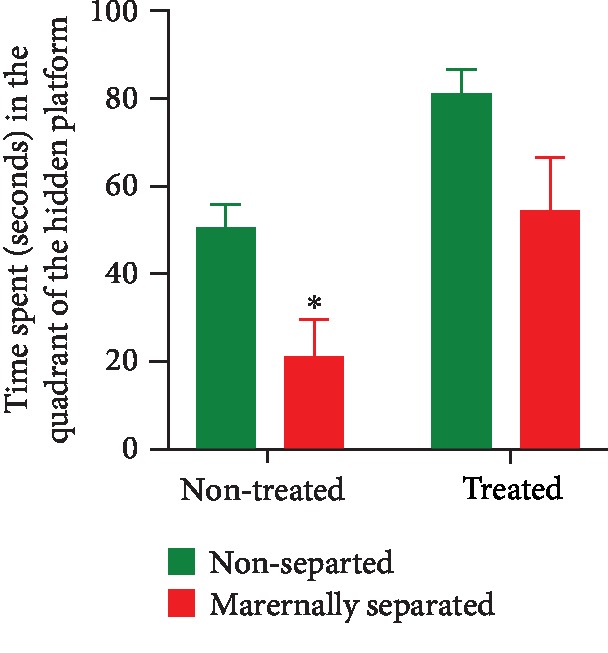
Effect of Fluvoxamine treatment on the memory retrieval of nonseparated (NS, NSF) and maternally separated (MS, MSF) rats measured as the time spent in the quadrant of the hidden platform of the MWM. Two-way ANOVA revealed a significant overall stress effect (*F*_(1, 36)_ = 11.60, *p* = 0.0016; ^∗^NS vs. MS, *p* = 0.0323, Tukey post hoc test) and an overall significant Fluvoxamine treatment effect (*F*_(1, 36)_ = 14.97, *p* = 0.0004). Data are presented as mean ± SEM (*n* = 10/group).

On PND 74, a two-way ANOVA revealed a significant overall stress effect (*p* = 0.0016) as maternally separated rats spent less time in the quadrant of the hidden platform when compared to nonseparated rats (NS vs. MS, *p* = 0.0323). A two-way ANOVA also revealed an overall significant Fluvoxamine treatment effect (*p* = 0.0004). Fluvoxamine treatment increased the time spent in the quadrant that had previously housed the hidden platform. Data are shown in Figure 8.

### 3.5. Lipid Peroxidation Levels

**Figure 9:**
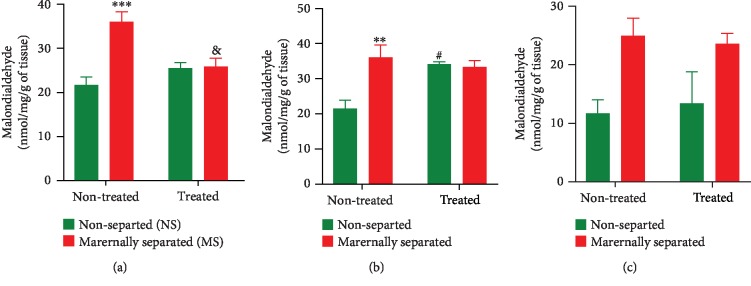
Effect of Fluvoxamine on malondialdehyde in the (a) striatum, (b) prefrontal cortex, and (c) hippocampus of nonseparated (NS, NSF) and maternally separated (MS, MSF) rats at PND 76. In the striatum (a), a two-way ANOVA revealed a significant interaction between stress and Fluvoxamine treatment (*F*_(1, 20)_ = 14.43, *p* = 0.0011; ^&^MS vs. MSF, *p* = 0.0042) and a stress effect (*F*_(1, 20)_ = 15.89, *p* = 0.0007; ^∗∗∗^NS vs. MS, *p* = 0.0001, Tukey post hoc test). *n* = 6/group. In the prefrontal cortex (b), a two-way ANOVA revealed a significant interaction between stress and Fluvoxamine treatment (*F*_(1, 8)_ = 11.18, *p* = 0.0102; ^#^NS vs. NSF, *p* = 0.0191) and a stress effect (*F*_(1, 8)_ = 8.995, *p* = 0.0171; ^∗∗^NS vs. MS, *p* = 0.0088, Tukey post hoc test). *n* = 3/group. In the hippocampus (c), a two-way ANOVA found no significant effect of Fluvoxamine treatment on MDA levels but an overall significant stress effect (*F*_(1, 8)_ = 11.78, *p* = 0.0089). *n* = 3/group. All data in this figure are expressed as mean ± SEM.

Malondialdehyde (MDA) levels were measured in the left hemisphere striatum, prefrontal cortex, and hippocampus of all rats on PND 76. In the striatum (Figure 9(a)), a two-way ANOVA revealed a significant interaction between stress and Fluvoxamine treatment (*p* = 0.0011) as the treatment decreased MDA levels in maternally separated rats (MS vs. MSF, *p* = 0.0042). A two-way ANOVA also revealed a stress effect (*p* = 0.0007) as MDA levels were significantly higher in maternally separated rats than in nonseparated rats (NS vs. MS, *p* = 0.0001). In the prefrontal cortex (Figure 9(b)), a two-way ANOVA revealed a significant interaction between stress and Fluvoxamine treatment (*p* = 0.0102) as the treatment increased MDA levels in nonseparated rats (NS vs. NSF, *p* = 0.0191). A two-way ANOVA also revealed a stress effect (*p* = 0.0171) as MDA levels were significantly higher in maternally separated rats than in nonseparated rats (NS vs. MS, *p* = 0.0088). In the hippocampus (Figure 9(c)), a two-way ANOVA showed an overall significant stress effect (*p* = 0.0089) on MDA levels. Hippocampal MDA levels were higher in maternally separated rats than in nonseparated rats. All data are presented in Figure 9.

### 3.6. Limb-Use Asymmetry (Cylinder Test)

Asymmetry of forelimb use was assessed in all rats on PND 58 and PND 74. Prior to 6-OHDA lesion (PND 58), no significant asymmetry was evident in the rats (data not shown).

**Figure 10:**
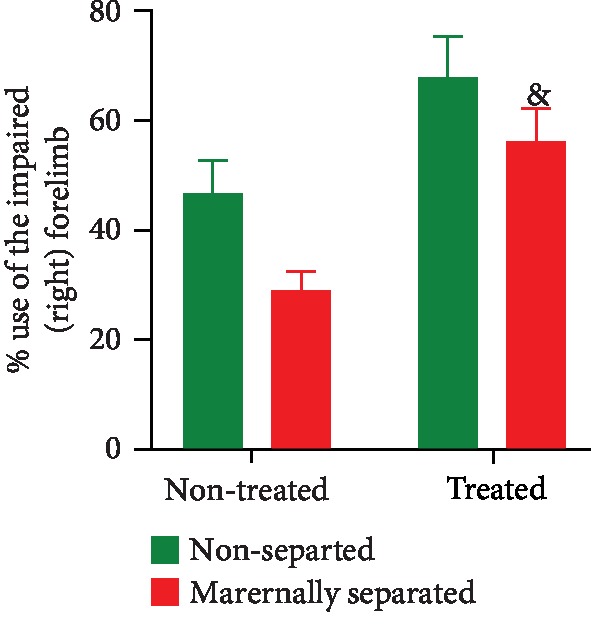
Effect of Fluvoxamine on the number of times the lesioned rat used the impaired (right) forelimb to touch the wall of the cylinder expressed as a percentage of the total number of times it touched the wall of the cylinder on PND 74. Two-way ANOVA revealed an overall significant effect of stress (*F*_(1, 36)_ = 6.196, *p* = 0.0176) and a Fluvoxamine treatment effect (*F*_(1, 36)_ = 16.65, *p* = 0.0002; ^&^MS vs. MSF, *p* = 0.0216, Tukey post hoc test). All groups were lesioned with 6-OHDA on PND 60. Values are expressed as mean ± SEM (*n* = 10/group).

On PND 74, a two-way ANOVA revealed a significant stress effect (*p* = 0.0176) and an overall significant Fluvoxamine treatment effect (*p* = 0.0002) as the treatment attenuated forelimb use asymmetry caused by the 6-OHDA lesion in all rats. Fluvoxamine treatment significantly increased the use of the impaired forelimb in maternally separated rats (MS vs. MSF, *p* = 0.0216). Data are presented in Figure 10.

### 3.7. Dopamine Levels

**Figure 11:**
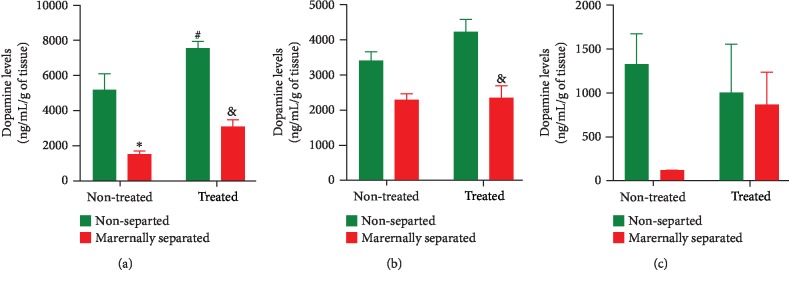
Effect of Fluvoxamine on dopamine in the (a) striatum, (b) prefrontal cortex, and (c) hippocampus of nonseparated (NS, NSF) and maternally separated (MS, MSF) rats at PND 76. In the striatum (a), a two-way ANOVA revealed a significant effect of maternal separation stress (*F*_(1, 20)_ = 56.31, *p* < 0.0001; ^∗∗∗^NS vs. MS, *p* = 0.0006; ^&^NSF vs. MSF, *p* < 0.0001, Tukey post hoc test) and an overall effect of Fluvoxamine treatment (*F*_(1, 20)_ = 13.05, *p* = 0.0017; ^#^NS vs. NSF, *p* = 0.0280, Tukey post hoc test). *n* = 6/group. In the prefrontal cortex (b), a two-way ANOVA revealed an overall stress effect (*F*_(1, 16)_ = 27.80, *p* < 0.0001; ^&^NSF vs. MSF, *p* = 0.0013, Tukey post hoc test). *n* = 5/group. In the hippocampus (c), a two-way ANOVA found no significant effect of Fluvoxamine treatment. *n* = 3/group. All data in this figure are expressed as mean ± SEM.

Dopamine (DA) levels were measured in the left hemisphere striatum, prefrontal cortex, and hippocampus of all rats on PND 76. In the striatum (Figure 11(a)), a two-way ANOVA revealed a stress effect (*p* < 0.0001) as DA was significantly lower in maternally separated rats when compared to nonseparated rats (NS vs. MS, *p* = 0.0006) and (NSF vs. MSF, *p* < 0.0001). There was also an overall Fluvoxamine treatment effect (*p* = 0.0017; (NS vs. NSF, *p* = 0.0280)) on DA levels in nonseparated rats. In the prefrontal cortex (Figure 11(b)), a two-way ANOVA revealed an overall effect of stress (*p* < 0.0001) in all treated rats (NSF vs. MSF, *p* = 0.0013). In the hippocampus (Figure 11(c)), although Fluvoxamine treatment tended to increase DA levels, a two-way ANOVA found no significant effect. All data are presented in Figure 11.

### 3.8. Serotonin Levels

**Figure 12:**
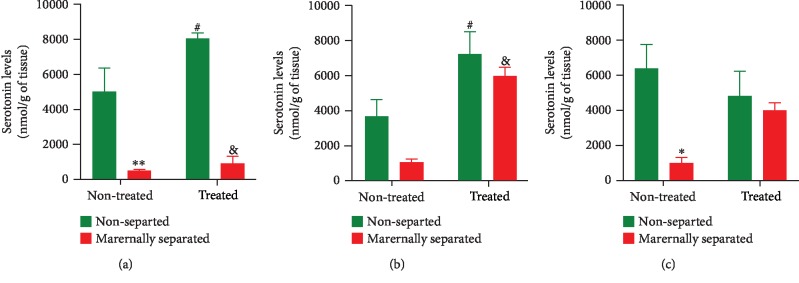
Effect of Fluvoxamine on serotonin in the (a) striatum, (b) prefrontal cortex, and (c) hippocampus of nonseparated (NS, NSF) and maternally separated (MS, MSF) rats at PND 76. In the striatum (a), a two-way ANOVA revealed a significant effect of maternal separation stress (*F*_(1, 20)_ = 63.28, *p* < 0.0001) and an overall effect of Fluvoxamine treatment (*F*_(1, 20)_ = 5.584, *p* = 0.0284; ^∗∗^NS vs. MS, *p* = 0.0016; ^&^NSF vs. MSF, *p* < 0.0001; ^#^NS vs. NSF, *p* = 0.0373, Tukey post hoc test). *n* = 6/group. In the prefrontal cortex (b), a two-way ANOVA revealed a significant effect of maternal separation stress (*F*_(1, 20)_ = 5.301, *p* = 0.0322) and an overall effect of Fluvoxamine treatment (*F*_(1, 20)_ = 25.41, *p* < 0.0001; ^#^NS vs. NSF, *p* = 0.0336; ^&^MS vs. MSF, *p* = 0.0026, Tukey post hoc test). *n* = 6/group. In the hippocampus (c), a two-way ANOVA revealed a significant effect of maternal separation stress on serotonin levels (*F*_(1, 8)_ = 9.232, *p* = 0.0161; ^∗^NS vs. MS, *p* = 0.0238, Tukey post hoc test). *n* = 3/group. All data in this figure are expressed as mean ± SEM.

Serotonin levels were measured in the left hemisphere striatum, prefrontal cortex, and hippocampus of all rats on PND 76. In the striatum (Figure 12(a)), a two-way ANOVA revealed a stress effect (*p* < 0.0001) as serotonin was significantly reduced in maternally separated rats when compared to nonseparated rats (NS vs. MS, *p* = 0.0016); (NSF vs. MSF, *p* < 0.0001). There was also an overall Fluvoxamine treatment effect (*p* = 0.0284) on serotonin levels in all treated rats (NS vs. NSF, *p* = 0.0373). In the prefrontal cortex (Figure 12(b)), a two-way ANOVA revealed a stress (*p* = 0.0322) and Fluvoxamine treatment (*p* < 0.0001) effect on serotonin levels which were increased in treated rats (NS vs. NSF, *p* = 0.0336 and MS vs. MSF, *p* = 0.0026). In the hippocampus (Figure 12(c)), a two-way ANOVA revealed a stress (*p* = 0.0161) effect as serotonin was significantly reduced in maternally separated rats when compared to nonseparated rats (NS vs. MS, *p* = 0.0238). All data are presented in Figure 12.

## 4. Discussion

This study is aimed at assessing the long-term effects of Fluvoxamine in managing anxiety/depressive-like symptoms and cognitive deficits and reducing motor dysfunctions in a parkinsonian rat model. Our objective was to evaluate the long-term indirect neuroprotective effect of Fluvoxamine on dopaminergic and serotonergic neurons which degenerate during PD.

Plasma corticosterone levels were measured to confirm the effectiveness of the stress protocol in dysregulating the HPA axis resulting in anxiety/depressive symptoms and cognitive deficits [27, 30, 36, 37, 56]. We found that maternally separated (MS) rats had higher corticosterone levels when compared to nonseparated (NS) rats in agreement with a study by Daniels et al. [27] who showed that stress elevates blood corticosterone levels. These results validated our stress protocol by showing that maternal separation induced a greater preference for the closed arms of the EPM for stressed animals, therefore, provided the evidence of anxiety-like behaviors as per studies by Mpofana et al. [12] and Daniels et al. [27]. The elevated blood corticosterone levels in maternally separated rats also confirmed depressive-like behaviors in the SPT and explained why maternally separated rats consumed less sucrose solution than nonseparated rats. These results suggested a long-lasting effect of our stress protocol in agreement with studies by Dalle et al. [19], Jayatissa et al. [57], and Daniels et al. [58]. Besides, given the hippocampus-dependent learning and memory, the long-lasting and elevated plasma corticosterone levels impaired learning and memory resulting in poor cognitive performance of maternally separated rats in the MWM test. This specific result agreed with previous studies and provided evidence that exposure to maternal separation impaired learning and memory in rats [32, 59–63].

On the other hand, in rats treated with Fluvoxamine, there was a reduction in plasma corticosterone levels in maternally separated (MSF) rats supporting the anxiolytic potential of the drug in rats exposed to chronic stress. This explained why we found that maternally separated treated rats spent more time in the open arms of the EPM than nonseparated treated (NSF) rats. The long-term treatment with Fluvoxamine produced noticeable behavioral relief in agreement with previous studies that showed that psychological disorders including anxiety and depression are relieved with prolonged and consistent antidepressant treatment [27, 64]. Fluvoxamine may have activated the HPA axis resulting in neuroendocrine responses via specific serotonergic neurons [65]. Since, for instance, increased corticosterone levels have been linked to anxiety/depressive-like signs [12, 27, 66, 67], Fluvoxamine's ability to decrease corticosterone levels in MSF rats confirmed its antidepressive-like effects as per studies by Hiemke and Hartter [66] and Irons [67]. This is why in the SPT, we found an increased sucrose solution preference in maternally separated rats treated with Fluvoxamine. Therefore, it is possible that, while depression altered the HPA axis, our SSRI drug (Fluvoxamine) restored the imbalance resulting in the observed reactivation of the hedonic value (sucrose preference) [40, 57, 68–72].

It was interesting to find that the decreased levels of plasma corticosterone induced a retrieval of spatial memory in all rats treated with Fluvoxamine (NSF and MSF) and tested in the MWM. Although this finding supports a previous study by Dalle et al. [45], this is the first to find Fluvoxamine's therapeutic effect in relieving cognitive impairment in a rat model of PD. Fluvoxamine seemed to prevent stress-induced cognitive impairment that usually results from impaired synaptic plasticity in the hippocampus. It is also possible that Fluvoxamine has memory-protective characteristics that blocked the adverse effects of chronic stress on the hippocampus-dependent learning and memory. Also, it has been previously shown that early maternal separation disturbs the HPA axis and leaves the brain more vulnerable to subsequent traumatic events later in life [1, 12]. Fluvoxamine may have antistress and memory-protective properties that do not interfere or alter the response of the HPA axis towards a stress event. For instance, Fluvoxamine may have accomplished its antistress effects by regulating corticosterone dysfunction which is known to have a critical role in the development of cognitive function or by normalizing stress-induced increase in glutamate in the hippocampus [73–76]. Long-term treatment with Fluvoxamine may, therefore, counteract the harmful effects of early-life stress that has life-long impacts on memory function. Here, we concluded that nonmotor symptoms (anxiety/depressive-like signs and cognitive deficits), which are reminiscent of chronic stress, are relieved by the long-term treatment with Fluvoxamine.

Chronic stress frequently involves oxidative brain damages [3]. To correlate changes in a marker of oxidative stress, neurotransmitter levels, and PD symptoms, malondialdehyde (MDA), dopamine (DA), and serotonin levels were evaluated in brain tissues. Our results showed that MDA levels were increased in the striatum, the prefrontal cortex, and the hippocampus of maternally separated (MS) rats in agreement with studies by Mabandla and Russell [29], who showed that 6-OHDA is an oxidative stress-induced neurodegeneration [77–82]. As our stereotaxic injection of 6-OHDA into the MFB (to disrupt striatal, prefrontal cortex, and hippocampal innervation) also provided a good measurement of oxidative damage in the brain, it was evident that the lesion contributed to the observed decreased use of the right forelimb (impaired) of MS rats in the cylinder test. These results agreed with previous studies by Mabandla and Russell [29] who showed that 6-OHDA lesion causes motor impairment and/or motor asymmetry which is characterized by a reduced use of the impaired forelimb resulting from dopaminergic neuron degeneration. However, it was interesting to find that Fluvoxamine treatment decreased MDA levels in the striatum (although with no significant effect on the prefrontal cortex and the hippocampus) of maternally separated and nonseparated rats (MSF and NSF). In fact, we found that all Fluvoxamine-treated rats did not show a preference for the use of the left forelimb (unimpaired) in the cylinder test suggesting a reduced toxic effect of the neurotoxin 6-OHDA due to the treatment. This major finding agreed with results from previous studies that showed that antidepressants can reduce MDA levels thereby significantly protecting striatal, prefrontal, and hippocampal cells and preventing neuronal loss associated with both chronic stress and 6-OHDA neurotoxicity [83–87]. We, therefore, postulated that Fluvoxamine possesses antioxidant properties (responsible for mopping up the free radicals generated by the autooxidation of 6-OHDA) and antitoxicity potential that reduced 6-OHDA neurotoxicity. Here, we concluded that chronic stress-induced oxidative stress was mitigated by Fluvoxamine treatment, lending support to the potential clinical use of Fluvoxamine as an antistress adaptogen with antioxidant efficacy for motor dysfunctions.

In PD, it is well documented that motor dysfunctions may result from dopamine (DA) depletion in the striatum, the prefrontal cortex, and the hippocampus [19, 30, 45, 88, 89]. This study only focused on how Fluvoxamine was indirectly implicated in the dopaminergic neuronal integrity in our PD model [90, 91]. Generally, we found reduced DA levels in the striatum and prefrontal cortex of maternally separated (MS) rats when compared to nonseparated (NS) rats. These results were predictable as previous studies showed that chronic stress (including maternal separation) leads to central dopaminergic denervation in brain areas implicated in movement, learning and memory, or motivation [1, 92–94]. In an animal model of PD, 6-OHDA selectively uptaken by dopaminergic neurons via DA transporters can exacerbate the harmful effects of chronic stress leading to apoptotic cell death in the striatum and related brain areas including the prefrontal cortex and hippocampus [12, 95, 96]. Our results, therefore, suggested that DA release was responsive to our combined model of chronic stress and 6-OHDA lesion.

On the other hand, we also found that Fluvoxamine treatment consistently preserved DA levels in the striatum and the prefrontal cortex of treated rats (NSF and MSF), a finding not reported elsewhere. This result shed light on the neuroprotective potential of Fluvoxamine which may have prevented 6-OHDA uptake. Besides, since SSRIs are involved in the release of both DA and serotonin in the striatal dopaminergic system that controls movements, the possible mechanism underlying the observed Fluvoxamine-mediated limb-use asymmetry is the proof that Fluvoxamine acted as a DA agonist following 6-OHDA lesion to counteract neuronal cell death [83, 84, 97]. This major finding suggests for the first time that Fluvoxamine may downregulate striatal DA transporters, potentially altering 6-OHDA uptake. For instance, Fluvoxamine treatment may have boosted the stimulation of striatal dopaminergic fibers from midbrain neurons to preserve pyramidal cell assemblies that consolidate motor, mood, or learning and memory functions. However, although DA deficiency in the striatum, the prefrontal cortex, and the hippocampus is expected to exacerbate motor deficits, learning and memory, and anxiety/depressive-like signs, we are the first to propose that Fluvoxamine may stimulate midbrain dopaminergic neurons to promote hippocampal network dynamics associated with long-term learning and memory performance. Indeed, the neurotoxin 6-OHDA could have reduced DA release potential at dopaminergic terminals in the striatum, prefrontal cortex, and hippocampus, but the long-term administration of Fluvoxamine may have preserved dopaminergic outputs hence the stable extracellular DA levels in all treated rats. At this stage, we acknowledge that the mechanism by which this occurs is entirely unclear and beyond the aim of this study.

Concerning the serotonergic system commonly affected in PD, we found that maternal separation stress and 6-OHDA lesion lowered serotonin levels in the striatum, the prefrontal cortex, and the hippocampus of maternally separated and nonseparated rats (MS and NS) [98, 99]. Our results concorded with the ones by Daniels et al. [27], Smolders et al. [100], and Mokler et al. [101] who demonstrated that stress can significantly alter serotonin levels resulting in executive functions involving the striatum, the prefrontal cortex, and the hippocampus being compromised. However, an increased level of serotonin in the striatum, prefrontal cortex, and hippocampus of all Fluvoxamine-treated rats (NSF and MSF) was also found. In the absence of comparative data, these results suggested that chronic stress combined with 6-OHDA lesion changed brain serotonin levels to initiate a sort of functional and/or structural compensatory mechanism associated with dopaminergic neurotransmission. The serotonergic dysfunction in PD nowadays relayed by many schools of thought was in some way associated with the development and/or complications of the motor (limb-use asymmetry) and the nonmotor (anxiety/depressive-like signs and cognitive deficits) symptoms observed in our study. Therefore, although chronic treatment with Fluvoxamine may attenuate parkinsonism by facilitating DA release to some brain regions, in a 6-OHDA-lesioned PD rat model with severe nigrostriatal dopaminergic neuron loss, striatal reuptake of Fluvoxamine-derived may occur via serotonin transporters. Taken together, here our serotonin analyses imply that many other factors played a role in a turnover of serotonin in maintaining normal brain function [102].

## 5. Conclusion

The long-term treatment with Fluvoxamine (expected to block serotonin transporters) may have instead “tricked” DA transporters such that dopaminergic innervation (in the striatum) was preserved. Fluvoxamine has more complex traffic which may not only be limited in restoring the serotonergic imbalance but may include adaptive changes that can result in the attenuation of parkinsonism. This study showed, for the first time, the beneficial effects of Fluvoxamine long-term treatment on monoamine levels in specific brain areas involved in PD pathogenesis. Our findings are consistent with the hypothesis that Fluvoxamine indirectly protects the syntheses and/or release of monoamines/neurotransmitters such as DA and serotonin to dampen dysfunction associated with parkinsonism. Together, these findings are of interest, considering that most neurodegenerative diseases including PD are associated with imbalance of DA and serotonin levels in the brain. Fluvoxamine may therefore be recommended to patients with PD at the earlier stage of the disease to avoid serotonergic/dopaminergic dysfunction that worsens parkinsonism.

## Data Availability

The data used to support the findings of this study are available from the corresponding author upon request.

## Abbreviations

6-OHDA: 6-Hydroxydopamine
DA: Dopamine
PD: Parkinson's disease
EPM: Elevated plus maze
SPT: Sucrose preference test
MWM: Morris water maze
NS: Nonseparated
NSF: Nonseparated treated with Fluvoxamine
MS: Maternally separated
MSF: Maternally separated treated with Fluvoxamine
PND: Postnatal day
TBARS: Thiobarbituric acid-reactive substances
MDA: Malondialdehyde
AP: Anterior-posterior
ML: Mediolateral
DV: Dura-ventral
MFB: Medial forebrain bundle
SSRI: Selective serotonin reuptake inhibitor.
